# Targeting the CCL2-CCR2 signaling axis in cancer metastasis

**DOI:** 10.18632/oncotarget.7376

**Published:** 2016-02-14

**Authors:** Su Yin Lim, Arseniy E. Yuzhalin, Alex N. Gordon-Weeks, Ruth J. Muschel

**Affiliations:** ^1^ CRUK/MRC Oxford Institute for Radiation Oncology, University of Oxford, Oxford, United Kingdom

**Keywords:** chemokines, cancer metastasis, cancer therapy, clinical trials

## Abstract

The CCL2-CCR2 signaling axis has generated increasing interest in recent years due to its association with the progression of cancer. Although first described as a chemotactic molecule with physiological roles in regulating inflammation, recent studies have revealed a pro-tumorigenic function for CCL2 in favoring cancer development and subsequent metastasis. CCL2 binds the cognate receptor CCR2, and together this signaling pair has been shown to have multiple pro-tumorigenic roles, from mediating tumor growth and angiogenesis to recruiting and usurping host stromal cells to support tumor progression. The importance of CCL2-CCR2 signaling has been further championed by the establishment of clinical trials targeting this signaling pair in solid and metastatic cancers. Here we review the roles of CCL2-CCR2 signaling in the development and progression of cancer metastasis. We further evaluate the outcome of several clinical trials targeting either CCL2 or CCR2, and discuss the prospects and challenges of manipulating CCL2-CCR2 interaction as a potential approach for combating metastatic disease.

## INTRODUCTION

Cancer metastasis remains the main cause of cancer related mortality and contributes to poor prognosis in the majority of cancer types. Metastases decrease the likelihood of survival; this is best represented by the significant decline in five year survival rates in prostate and breast cancer patients with metastasis upon presentation [1]. Surgical intervention and other conventional treatments have limited efficacy once cancer cells have spread to distant sites, owing to the heterogeneity and aggressiveness of the disease, high chance of recurrence and the difficulty in targeting multiple sites after metastatic spread. Research efforts have been directed at interfering with key mediators important in metastatic development as alternative strategies for cancer treatment. Over the past few decades, distinct chemokine-receptor signaling pathways have emerged as attractive targets for therapy due to their key roles in the metastatic process.

Chemokines and their receptors mediate acute inflammation and were initially described in the context of their chemoattractant function for leukocytes; chemokines, induced at sites of inflammation, provide directional cues during migration of leukocytes to damaged or infected tissues [2, 3]. However, elevated expression of chemokines, leading to alterations in chemokine-receptor signaling can contribute to chronic inflammation and malignancy [4, 5]. Cancer cells and host stromal cells in the tumor microenvironment including endothelial cells, fibroblasts, mesenchymal stem cells and infiltrating leukocytes produce a wide range of chemokines that exert numerous biological functions during tumor progression and metastasis [6, 7]. Of these, CCL2 together with its cognate receptor CCR2 have been shown to play key roles in cancer metastasis by sustaining cancer cell proliferation and survival, stimulating cancer cell migration and invasion, and inducing deleterious inflammation and angiogenesis. Here we provide an overview of CCL2-CCR2 signaling during the metastatic process, and draw upon experimental and clinical studies to highlight their significant contributions to metastatic development and progression. Finally, we discuss the relevance and efficacy of targeting this signaling pair as a means of therapeutic intervention.

## BIOLOGY OF CCL2 AND CCR2

CCL2 belongs to a group of low molecular weight cytokines with chemoattractant activity, collectively known as chemokines. As a prototypic chemokine, CCL2 orchestrates immune cell recruitment to specific sites, and is expressed constitutively for homeostatic functions such as regulating lymphocyte trafficking from blood to lymph nodes, and is induced during inflammatory responses when leukocytes are required for tissue defense and repair [3]. CCL2 is expressed by a wide range of cells including endothelial, epithelial, myeloid and smooth muscle cells and fibroblasts, either constitutively or after induction, and is a potent chemoattractant for monocytes, basophils, T lymphocytes and NK cells [8].

CCL2 was initially categorized as monocyte chemotactic protein 1 (MCP-1) due to its structural similarity with other MCPs, including MCP-2 (CCL8), MCP-3 (CCL7), MCP-4 (CCL13) and MCP-5 (CCL12). These MCPs share high sequence homology and highly conserved secondary structures of two adjacent N-terminal cysteine residues that are important for receptor binding [9]. Binding of a chemokine to its cognate receptor is required to trigger signal transduction pathways and exert biological effects such as chemotaxis. These cell surface G protein-coupled receptors are characterized by an N-terminal extracellular domain, 7 conserved transmembrane domains linked by three intracellular and extracellular loops, and a serine/threonine-rich C-terminal intracellular domain, the latter of which is coupled to a heterotrimeric G-protein for signal transduction.

CCL2 preferentially binds the CCR2 receptor, which is expressed in various tissues including blood, brain, heart, kidney, liver, lung, ovary, pancreas, spinal cord, spleen and thymus. CCR2 is expressed as two isoforms due to alternative splicing: CCR2A and CCR2B, which differ by 50 base pair in the C-terminal domain [10]. CCR2B is the predominant isoform of CCR2 surface receptors, highly expressed by monocytes and NK cells, and accounting for 90% of all CCR2 expressed [11, 12]. CCR2A is expressed by a small subset of mononuclear and smooth muscle cells [13]. It is important to note that binding of CCL2 to the CCR2A isoform induces different biological responses from binding to the CCR2B isoform, as demonstrated in Jurkat T cells [14]. Cell-specific expression of the CCR2 isoforms may serve as a means of functional regulation.

Apart from CCR2, CCL2 can also bind other chemokine receptors. CCL2 has been reported to bind CCR4 on cytotoxic T lymphocytes, resulting in their recruitment to melanoma cells [15] thus implicating an immune-mediated protective role in cancer. However, CCL2-CCR4 signaling also recruits T regulatory cells to tumor sites, which may result in cytotoxic T cell suppression [16]. CCL2 has also been shown to bind two atypical receptors, ACKR1 and ACKR2. These atypical receptors do not signal through G proteins and are regarded as decoys or scavenger receptors as they lack chemotactic activity. Scavenger receptors are likely to compete for CCL2 binding with other conventional G-protein coupled chemokine receptors [17], enabling them to modulate free CCL2 levels [18].

CCR2 is a promiscuous receptor that binds other chemokines, particularly other MCPs including MCP-2 (CCL8), MCP-3 (CCL7) and MCP-4 (CCL13) consistent with their structural similarity to CCL2. This flexibility in chemokine-receptor interaction may lead to different biological outcomes, depending on the particular chemokine and receptor pair engaged [19], or may produce similar effects, suggesting redundancies in chemokine function. For example, binding of either CCL7 or CCL2 to CCR2 can stimulate monocyte emigration from the bone marrow to sites of metastasis [20]. Likewise, binding or either CCL8 or CCL2 to CCR2 on colorectal cancer cells can provoke a similar increase in migration and invasion [21]. However, distinct effects have also been noted; binding of CCL8 to CCR8 promotes the recruitment of inflammatory Th2 cells whereas its binding to CCR2 did not generate the same response [22].

Because CCL2 and CCR2 bind other receptors and ligands respectively, understanding the specific effects and implications of CCL2-CCR2 signaling has been challenging. However, the CCL2-CCR2 pairing appears to be the prevalent interaction *in vivo* as mice deficient in CCL2 share similar phenotypes to those deficient in CCR2 [23, 24].

## THE ROLE OF CCL2-CCR2 SIGNALING IN THE METASTATIC CASCADE

**Figure 1:**
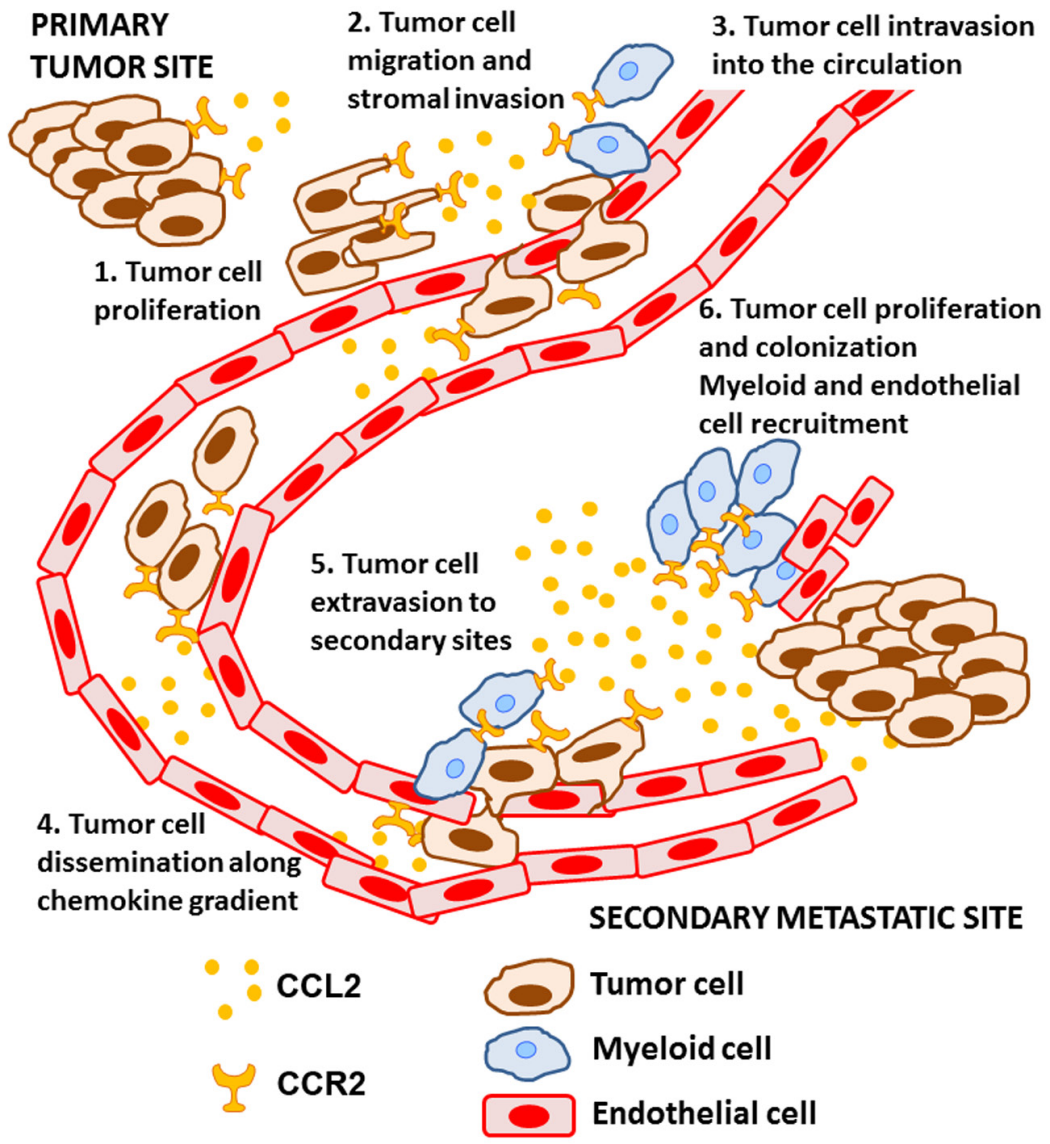
The role of CCL2-CCR2 signaling during the metastatic process CCL2 is expressed by cancer and stromal cells in the tumor microenvironment and, 1) induces tumor cell proliferation at the primary tumor site and 2) stimulates tumor cell migration and invasion into the surrounding extracellular matrix. CCL2 subsequently 3) promotes tumor cell intravasation into the circulation, likely by recruiting host myeloid cells to facilitate this process. Once in the circulation, CCL2 may 4) direct the dissemination of cancer cells along a chemotactic gradient towards the metastatic site. Trapping of tumor cells in small capillaries initiates 5) tumor cell extravasation, which is further supported by CCR2^+^ myeloid cells and the CCR2^+^ endothelium. Finally, CCL2 6) promotes tumor growth at the metastatic site, and tumor colonization by recruiting additional myeloid and endothelial cells.

Metastasis is a multistep process that involves invasion of cancer cells into surrounding tissues, followed by intravasation into lymph or blood vessels whereupon tumor cells disseminate until their arrest and extravasation into secondary sites. At secondary sites, cancer cells must adapt to their new microenvironment in order to proliferate and form metastatic outgrowths; this latter stage is often referred to as metastatic colonization [25–27]. Cancer cells are vulnerable to death at any of these steps and will require intrinsic and extrinsic responses to enable their survival. CCL2-CCR2 signaling is especially important for successful metastasis, and has been shown to be involved in both the early and late steps of the metastatic process in experimental models. CCL2 can be produced by both cancer and stromal cells in the tumor microenvironment, exerting direct effects on cancer cells and functioning indirectly by recruiting host stromal cells with pro-tumorigenic activities during metastasis (Figure 1).

During the early stages of metastasis, cancer cells acquire a migratory and invasive phenotype, which allows them to break down surrounding extracellular matrix (ECM), invade neighboring tissues and move towards the blood or lymph vessels. CCL2 contributes towards this initial stage, guiding cancer cell migration by interacting with the CCR2 receptor expressed on tumor cells [28, 29]. Additionally, CCL2 induces expression of metalloproteinases MMP2 and MMP9 in cancer cells, leading to increased invasion [30, 31]. Following migration and invasion, intravasation of cancer cells into the circulation is required to allow for metastatic dissemination. Wyckoff *et al.* demonstrated that this process may necessitate cancer cell interaction with tumor associated macrophages (TAMs) [32]. Extravasation of cancer cells out of the circulation to secondary sites is also dependent on association with host stromal cells including TAMs [33] and bone marrow endothelial cells [34]. Because CCL2 is a potent chemoattractant for TAMs, it may indirectly promote the intravasation and extravasation process of cancer cells.

Arguably, the most challenging step of the metastatic cascade occurs after cancer cells have successfully extravasated from the circulation. At secondary sites, most cancer cells die, enter a homeostatic balance of cell proliferation and apoptosis that prevent further progression, or remain quiescent [35]. This state of dormancy is in keeping with clinical observations that many patients with melanoma, or breast and prostate carcinomas develop metastatic relapse years after the initial diagnosis or treatment. Breast cancer recurrences in particular are at times detected decades after remission [36, 37]. Tumor dormancy occurs in response to delayed adaptation to the new microenvironment and may be a coping mechanism whilst cancer cells accumulate sufficient resources and/or properties for successful proliferation and colonization. For example, cancer cells may need to develop means of evading immunosurveillance (to prevent immune dormancy), circumventing cell cycle arrest (to prevent cellular dormancy) and/or triggering an angiogenic switch (to prevent mass dormancy) for successful metastatic formation [38–40]. Although there has been little evidence linking CCL2 or CCR2 to cell cycle arrest, CCL2-CCR2 signaling has been shown to recruit myeloid cells such as TAMs to incite an angiogenic switch [41, 42], and myeloid-derived suppressor cells (MDSCs) to suppress and evade immune-mediated killing [43].

CCL2 has additional roles during the later stages of metastasis. Cancer cells can usurp the leukocyte trafficking mechanism to preferentially home to specific organs [44, 45]. Hence, in addition to leukocytes, CCL2 can also attract cancer cells to secondary sites, although this process is more complex and may require interactions with other hematopoietic cell types [46]. CCL2 can further stimulate cell proliferation and enhance survival once cancer cells have been recruited to metastatic sites [47, 48].

CCL2, along with other CXC chemokines such as CXCL1, CXCL2, CXCL3, CXCL5, CXCL6, CXCL7, and CXCL8 promote recruitment, migration and proliferation of endothelial cells. Both mouse and human endothelial cells express CCR2 and can be recruited to the metastatic microenvironment in response to CCL2. Additionally, CCL2-CCR2 signaling as a mediator of neovascularization has been previously demonstrated in several *in vitro* and *in vivo* models of angiogenesis [49–52].

Apart from direct effects on tumor and endothelial cells, CCL2 also recruits various immune cell subsets including monocytes [53] and macrophages [54] to site of metastasis, and mediates differentiation and polarization of these cell types [55, 56]. Immune cell polarization can result in distinct, if not opposing, functional properties, and may ultimately tip the balance between tumor promotion or inhibition. For example, macrophages polarized towards an M2 phenotype are immunosuppressive, and may enhance tumor cell survival by dampening immune surveillance and attack [55]. Similarly, CCL2 is involved in T cell differentiation and polarization [57], and has been shown to regulate Th2 polarization towards a more immunosuppressive T regulatory phenotype *in vitro* and in mouse models [58–61].

It is evident that CCL2-CCR2 signaling has multiple key functions, on both cancer and stromal cells (summarized in Table 1), altogether of which appear to predominately favor metastatic development and progression. In the following sections, we highlight effects of CCL2/CCR2 signaling in several experimental studies on metastatic cancers, with a particular focus on prostate, breast and colorectal cancers.

**Table 1:** Effects of CCL2-CCR2 signaling on cancer and stromal cells in the tumor microenvironment

CCL2-CCR2 signaling on cancer cells		
Cancer cell types	Effects	References
Breast cancer cells MCF-7	Stimulates cell migration	[102]
Breast cancer cells PyVmT, 4T1, MCF-7, MDA-MB-231	Stimulates cell survival and motility	[103]
Breast cancer cells MDA-MB-231 and MCF-7	Stimulates cell attachment to lymphatic endothelial cells	[104]
Prostate cancer cells PC-3, LNCaP, DU145	Stimulates cell adhesion and invasion	[105]
Prostate cancer cells PC-3	Stimulates cell proliferation and invasion	[52]
Prostate cancer cells PC3, DU145, and LNCaP	Stimulates cell migration and invasion	[48]
Prostate cancer cells PC-3 and VCaP	Stimulates proliferation and migration	[47]
Prostate cancer cells PC-3	Stimulates invasion and transendothelial cell migration	[30]
Glioblastoma cells T98G, and U87MG	Simulates cell migration and invasion	[106]
Ovarian cancer cells SKOV-3	Stimulates cell invasion and adhesion	[107]
Bladder cancer cells SV-HUC-1, RT4, TSGH8301, and J82	Stimulates cell migration and tumorigenicity	[24]
Chondrosarcoma cells JJ012	Stimulates cell migration	[27]
**CCL2-CCR2 signaling on stromal cells**		
**Stromal cell types**	**Effects**	**References**
Fibroblasts	Stimulates anti-fibrotic effects, survival, and adhesion	[108–110]
Myeloid-derived suppressor cells	Stimulates accumulation in tumors and affects immunosuppressive features	[66, 111]
Macrophages	Stimulates recruitment and infiltration; promotes normal peritoneal macrophages to acquire features of TAMs	[44, 54, 64, 112–114]
Monocytes	Stimulates maturation into macrophages	[44, 112]
Neutrophils	Stimulates recruitment	[115, 116]
Osteoclasts	Stimulates osteoclast formation and differentiation	[52, 117]
Stem cells	Stimulates migration and enhances pluripotency	[118, 119]
NK cells	Stimulates migration	[120, 121]
T cells	Stimulates migration, promotes Th2 polarization and negatively regulates Th1 response	[46, 122]
CD4+ Th17 T cells	Inhibits proliferation and activity	[123, 124]

## EFFECTS OF CCL2-CCR2 SIGNALING IN METASTATIC CANCERS: EXPERIMENTAL EVIDENCE

### Prostate cancer

CCL2 is predominantly expressed by endothelial cells in the prostate tumor microenvironment and by prostate cancer cell lines such as PC3 and LnCaP. CCL2 directly stimulates PC3 and VCaP prostate cancer cell proliferation and migration *via* activation of the PI3K/Akt signaling pathway and activation of Rac GTPase, respectively [62]. A separate, but similar study reported that CCL2 promoted PC3, LnCaP and DU145 prostate cancer cell migration in a PKC-dependent manner by upregulation of αvβ3 integrin expression on cancer cells [63]. In addition to proliferation and migration, CCL2 protects prostate cancer cells from autophagic death by activating the PI3K/Akt/survivin pathway [47].

In addition to direct effects on cancer cells, CCL2 also mediates stromal cell responses in the prostate tumor microenvironment. CCL2 blockade using CCL2 neutralizing antibodies (anti-mouse CCL2/JE C1142) in SCID mice after subcutaneous injection of VCaP cells suppressed tumor growth, decreased CD68^+^ macrophage infiltration, and led to reduced tumor-associated microvasculature [64]. However, although CCL2 has been shown to have direct angiogenic effects on HUVEC and HDMVEC, in this context, CCL2 was shown to stimulate tumor cells to upregulate expression of angiogenic factors such as VEGF [65].

Both tumor- and stromal-derived CCL2 contribute towards prostate tumor progression. Inhibition of tumor-derived CCL2 *via* administration of anti-human CCL2 blocking antibody (CNTO888) resulted in reduced tumor burden, albeit not to the extent as inhibition of stromal-derived CCL2 using the anti-mouse CCL2/JE antibodies. Additionally, use of both tumor and stromal-specific inhibitory CCL2 antibodies were not as effective compared to single-agent docetaxel, the standard treatment for hormone-refractory metastatic prostate cancer. However, when used in combination with docetaxel, a dramatic regression in tumor burden was observed; tumor regression was only maintained with repeated antibody administration as discontinuation of treatment resulted in tumor re-growth [66].

Prostate cancers typically metastasize to the bone, and inhibition of both tumor and stromal-derived CCL2 had striking effects in decreasing metastatic bone lesions [65, 66]. In accordance with this finding, CCL2 overexpression in PC3 cells increased metastatic bone lesions, with concurrent increases in the number of activated osteoclasts and infiltrating macrophages [54]. Lu *et al*. further showed that CCL2 may mediate bone resorption by inducing differentiation of osteoclast-like cells [67]. Targeting CCL2 using the anti-human CCL2 (CNTO888) antibodies, either alone or in combination with docetaxel, has also been shown to inhibit PCa prostate cancer cell growth in the bone [68]. Overall, these studies highlight the multifaceted roles of CCL2 in prostate cancer and suggest that both tumor and stromal derived CCL2 have direct and indirect effects in promoting prostate tumor growth and associated bone metastasis.

### Breast cancer

CCL2 is highly expressed by various breast cancer cell lines including 4T1, 4T07 and 67NR, as well as by both the hematopoietic and non-hematopoietic cells in the tumor stroma [69, 70]. CCL2 was shown to promote the migration of mammary carcinoma cell lines MCF-7, T47D and ZR-75-1 [71], and enhanced the migration and survival of 4T1, PyVmT, MDA-MD-231 and MCF-7 cells *via* activation of Smad3 and p42/44 MAPK signaling [72].

In addition to its direct effects on tumor cells, tumor and stromal-derived CCL2 also stimulated metastatic progression by recruiting different subsets of myeloid cells including TAMs [73], metastasis associated macrophages (MAMs) [74] and inflammatory monocytes [75]. Stromal CCL2 prompted macrophage infiltration to mammary tumor xenografts and its blockade using neutralizing antibody significantly reduced macrophage recruitment and tumor growth [76]. Similarly, CCL2 deficiency in the tumor stroma led to decreased macrophage recruitment and angiogenesis, consequently reducing incidence of lung metastases [69]. The contribution of stromal CCL2 may outweigh that of tumor-derived CCL2 as inhibition of stromal CCL2 alone significantly reduced primary tumor growth and distant lung metastases in orthotopic breast cancer models [77].

Qian *et al.* demonstrated that total blockade of CCL2 (both stromal- and tumor-derived) inhibited recruitment of CCR2^+^ inflammatory monocytes and prolonged survival of tumor-bearing mice; depletion of tumor-derived CCL2 alone was sufficient to inhibit metastatic seeding in the lung. The authors further implicated CCL2-mediated recruitment of CCR2^+^ monocytes as essential in enhancing tumor cell extravasation [53]. CCR2^+^ monocytes differentiate into CCR2^+^ MAMs, and in a subsequent study, the authors showed that CCL3 secretion by CCR2^+^ MAMs prolonged their retention in metastatic sites [74]. Overall these studies indicate that CCL2-CCR2 signaling is an important initial event leading to pulmonary metastasis of breast cancer.

In breast cancers, the recruitment of macrophages by CCL2 may also lead to extensive neovascularization [78] suggesting that tumor infiltrating myeloid cells may directly contribute to the angiogenic process although the mechanisms for this are not yet well defined. CCL2 from immortalized human mammary epithelial cells was recently shown to induce angiogenesis *via* upregulation of the transcription factor Twist1 [41] and presence of both CCL2 and macrophage infiltration was necessary for Twist1-driven angiogenesis, suggesting that CCL2 may recruit macrophages and thus, indirectly upregulate Twist1.

Another interesting study by Tsuyada *et al*. [79] revealed that human cancer associated fibroblasts (CAFs) co-cultured with human breast cancer cell lines BT474, MDA-MB-361 and MCF7 upregulated CCL2 in the cancer cells. Upregulation of CCL2 provoked a stem cell-like mammosphere-forming phenotype in the breast cancer cells. Furthermore, CCL2 enhanced self-renewal and expansion of the sphere-initiating cancer stem cells through STAT3 and NOTCH1 signaling pathways, suggesting that CCL2 may confer a more aggressive stem cell-like tumor phenotype. Overall, these findings strongly indicate that CCL2-CCR2 signaling in cancer and stromal cells is a major factor in the promotion of breast cancer growth and metastasis.

### Colorectal cancer

CCL2 signaling was shown to be an important precursor to colorectal cancer development as its absence in CCL2^−/−^ mice blocked neoplastic progression from dysplasia to adenocarcinoma. At later stages, CCL2 stimulated the accumulation of myeloid-derived suppressor cells (MDSCs) in colonic tumors, and enhanced MDSC-mediated suppression of T cells in a STAT3-dependent manner. Furthermore, CCL2 blocking antibodies decreased tumor and MDSC numbers, suggesting dual functions of CCL2 on both cancer and stromal cells [80].

Colorectal cancers typically metastasize to the liver although spread to the lung, peritoneum and other organs are also observed. Our group recently demonstrated the importance of CCL2-CCR2 signaling in an experimental mouse model of liver metastasis. CCL2, highly expressed by colorectal cancer cells, promoted the recruitment of a CD11b^+^ myeloid population expressing CCR2 from the bone marrow to the metastatic site in the liver. Depletion of CD11b^+^CCR2^+^ myeloid cells in the transgenic CD11b-DTR mouse model by diphtheria toxin administration markedly decreased metastatic growth and incidence [81].

Interestingly, in this system, attempts to inhibit CCL2 or CCR2 led to unexpected consequences. Inhibition of CCL2 using a functional blocking antibody, which targets both stromal and tumor cell-derived CCL2, did not alter myeloid cell recruitment and had little effect on metastatic tumor burden. This is in contrast to CCL2 inhibition in a breast cancer model, where Qian *et al.* showed decreased recruitment of CCR2^+^ inflammatory monocytes and reduced metastatic burden [53]. These differences may be model or organ specific but it is also likely that dosage of the CCL2 blocking antibody was not sufficient to inhibit CCL2-mediated effects. In support of this, serum CCL2 levels in mice were higher after treatment with the blocking antibody [81], suggesting a compensatory increase in CCL2 following its blockade.

In the experimental liver metastasis mouse model, knockdown of CCL2 expression in cancer cells caused a transient reduction in myeloid cell recruitment and a temporary delay in metastatic tumor growth, but these differences were not apparent 2 weeks after tumor cell injection [81]. The effect of CCR2 blockade was also disappointing; myeloid cell recruitment and metastatic tumor growth in the liver were reduced only to a small extent in CCR2 knockout mice, suggesting that CCL2 may bind other chemokine receptors apart from CCR2 to favor metastatic progression [81].

In addition to myeloid cell recruitment, tumor derived CCL2 has been shown to have direct effects on the tumor vasculature. CCL2 binding to CCR2^+^ endothelium enhanced vascular permeability in a p38/MAPK dependent manner, which in turn increased colon cancer cell extravasation and metastasis to the lungs [82].

### Other cancers

Blockade of CCL2 using inhibitory antibodies suppressed growth of pancreatic cancer cells PANC-1 and BXPC3 in culture and in subcutaneous tumor models [83]. Blockade of the CCR2 receptor, on the other hand, inhibited infiltration of immunosuppressive CCR2^+^ myeloid cells in a pancreatic cancer mouse model, resulting in significant reduction in subcutaneous tumor growth, as well as incidence of liver metastases [84]. Inhibition of both tumor and stromal derived CCL2 in a mouse glioma and human glioma xenograft model prolonged survival of mice with a corresponding decrease in TAMs and MDSCs [85].

CCL2-CCR2 signaling has also been implicated in metastasis in other cancers. In chondrosarcoma, which preferentially metastasizes to the lungs, CCL2 promoted migration and invasion of chondrosarcoma cells, likely by inducing MMP9 expression *via* Ras/Raf-1 and NF-κB activation [31]. In ectopic and orthotopic xenograft models of gastric cancer, CCL2 overexpression in the gastric cancer cell line TMK-1 significantly increased tumorigenicity and induced metastasis to lymph nodes. Tumors formed by CCL2 overexpressing cancer cells were also more angiogenic compared to controls [86]. Similar findings were reported for bladder cancer whereby functional blockade of CCL2 or CCR2 inhibited migration of T24, J82 and SV-HUC-1 cancer cells, reduced tumorigenicity [28] and suppressed development of lung metastases through the inhibition of versican [87]. CCL2 inhibition also reduced macrophage infiltration to the lung, which may additionally contribute to inhibition of metastasis formation [87].

CCL2 was also shown to mediate the growth and proinvasive capacity of metastatic melanoma cells [88]. Targeting CCL2 using neutralizing antibodies or with BRAF inhibitors, which suppressed *CCL2* gene expression, resulted in a marked inhibition of tumor growth in mouse models of metastatic melanoma [89]. In addition to having direct effects, CCL2 expressed by fibrocytes was implicated in recruitment of Ly6C monocytes, predisposing B16F10 cells to metastasize to the lung [90]. In agreement, other studies have shown that overexpression of CCL2 in melanoma cells enhanced vascular permeability and angiogenesis, and increased recruitment of mononuclear cells [91] and M2 polarized macrophages [92]. However, CCL2 may also have opposing anti-tumorigenic functions in melanoma progression. CCL2 expressed by melanoma cells has been shown to recruit cytotoxic T lymphocytes *via* binding to the CCR4 receptor on the lymphocytes [15]. Indeed, CCL2 overexpression in B16 melanoma cells enhanced Th2 cytokine production and reduced metastatic pulmonary tumor growth in wildtype C57BL/6 mice but not in nude mice with deficient adaptive immune responses [93], implying that T cells may be involved in CCL2-mediated anti-metastatic effects. Hence, effects of CCL2 may be dependent on specific receptor binding (CCR2 compared to CCR4) and also dependent on the type of effector cells involved.

## TARGETING CCL2-CCR2 SIGNALING IN THE CLINICAL SETTING

Elevated levels of CCL2 have been reported in patients with breast, colorectal, prostate, melanoma, gastric and ovarian cancers, and was often correlated with disease progression [94].

CCL2 was identified from meta analyses of gene expression datasets on prostate tumorigenesis as a primary driver of prostate cancer development [95] consistent with the experimental findings summarized above. Indeed, CCL2 appears to be a promising biomarker as it was overexpressed in tissue and serum samples of prostate cancer patients [96], and its expression correlated with Gleason score and pathologic state [97], and a more aggressive phenotype [48]. In ovarian cancer, CCL2 levels significantly correlated with histological grade and CCL2 was suggested as a differentiation marker between benign ovarian cysts and ovarian cancer [98].

CCL2 is overexpressed at tumor sites, malignant pleural effusions, and sera of breast cancer patients, and its increased levels in the tumor microenvironment and circulation were associated with disease progression, early relapse, tumor grade and aggressiveness, and poor prognosis [99]. Elevated CCL2 levels also inversely correlated with relapse-free survival, and predicted advanced tumor stage and lymph node involvement in breast cancer patients [100].

In patients with gastric cancer, elevated serum and intratumoral CCL2 levels significantly correlated with lymph node metastasis [101, 102]. Gastric cancer patients with high CCL2 expression also had a lower overall survival rate, suggesting CCL2 to be a prognostic marker for gastric cancer [103]. Similarly, in patients with primary and metastatic colorectal cancer, CCL2 levels were elevated in serum, and increased with progressive Dukes' stages [81] and neoplastic progression [80]. CCL2 was further implicated as a prognostic marker and an independent predictor of liver metastasis in colorectal cancer patients [104].

Taken together with the many experimental studies supporting an important pro-tumorigenic role for CCL2-CCR2 signaling, several clinical trials have been established to investigate the safety and efficacy of CCL2 or CCR2 blockade as means of therapeutic intervention.

### CCL2 inhibition (CNTO888) in solid tumors

**Figure 2:**
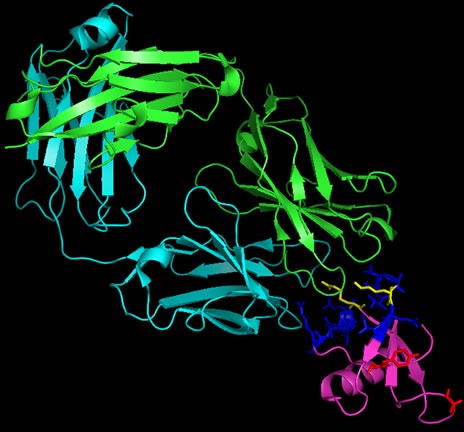
Ribbon representation of CNTO888 in complex with CCL2 CCL2, represented in magenta, comprises an anti-parallel 3-stranded β-sheet and a C-terminal α helix. The light chain of the CNTO888 antibody is shown in cyan whilst the heavy chain is shown in green. The epitope (on CCL2) recognized by CNTO888 (residues 18-24 and 45-51) is shown in blue whilst those important in CCR2 binding (Tyr13, Arg24, Lys35 and Lys49) is shown in red. The CNTO888 and CCR2 receptor epitope both include Arg24 and Lys49, shown in yellow. Figure was generated with PyMoL [116] using crystal structure of CNTO888 and CCL2 complex (PDB ID: 4DN4).

Carlumab, previously known as CNTO888, is a human IgG1_k_ monoclonal antibody developed by Centocor, that binds CCL2 with high affinity (disassociation constant of 15 pM), consequently inhibiting CCL2 binding to the CCR2 receptor [105]. CNTO888 interaction is also highly specific as it does not bind other mouse or human CC chemokines despite sharing high sequence homology. Analysis of the crystal structure of CNTO888 in complex with CCL2 [105] revealed residues 18-24 and 45-51 on CCL2 to be important for this interaction (Figure 2). Furthermore, comparison of the CCR2 receptor binding site indicated common epitopes shared by the inhibitory antibody and the CCR2 receptor, particularly Arg24 and Lys49 (Figure 2). Hence, the mode of action of CNTO888 is by direct competition with the CCR2 receptor binding site.

CCL2 inhibition was evaluated in solid tumors for safety and pharmacokinetic/pharmacodynamic parameters, in which CNTO888 was administered either alone or in combination with other standard of care chemotherapies. A phase 1 clinical trial (NCT00537368) was conducted in 44 patients with advanced solid tumors refractory to conventional treatments. As secondary measures, anti-tumor responses based on PSA and cancer antigen 125 (CA125) levels were also monitored. CNTO888 was administered intravenously at a starting dose of 0.3 mg/kg to a maximum planned dose of 15 mg/kg.

CNTO888 was well tolerated in these patients with less than 37% experiencing adverse events, all of which were mild in severity. These adverse events were likely related to CNTO888 administration as they were resolved following treatment discontinuation. However, CCL2 was only transiently suppressed following the first CNTO888 administration and subsequent dosing saw increased free CCL2 concentration to levels beyond pretreatment baseline values. None of the patients had an objective anti-tumor response although 4 maintained stable disease for 10.5 (patient with ovarian cancer), 5 (patient with prostate cancer), 7.2 (patient with ocular melanoma) and 15.7 (patient with neuroendocrine tumor) months [106].

In another phase 1 clinical trial (NCT01204996), the safety and pharmacokinetic/pharmacodynamics profile of CNTO888 in combination with 4 standard of care chemotherapies was assessed in 53 patients with solid tumors. These patients were divided into 4 treatment arms receiving CNTO888 with either docetaxel, gemcitabine, paclitaxel-carboplatin, or pegylated liposomal doxorubicin hydrochloride. Not unexpectedly given the combination treatments, more adverse events were observed with up to 93% of patients from each group experiencing hematological (ie neutropenia, thrombocytopenia, anemia) and non-hematological (ie nausea, stomatitis, fatigue) complications. Addition of CNTO888 to the treatment regime did not affect the pharmacokinetics of standard chemotherapies but the treatments also did not cause prolonged inhibition of CCL2; serum CCL2 initially declined but gradually increased. Despite the lack of CCL2 inhibition, 3 patients showed a 30% decrease in urinary cross-linked N-telopeptide of type I collagen (uNTX) values, used as a biomarker of bone resorption. Furthermore, 18 patients from all treatment arms showed stable disease ranging from 2 to 10.8 months and one patient achieved partial response. However, these objective responses were attributed to effects of the standard chemotherapies [107].

Follow-up pharmacodynamic analysis from these trials indicated that, contrary to its high binding affinity reported from *in vitro* studies, CNTO888 disassociation constant was much higher (2.4 nM) in patients, suggesting that it bound free CCL2 with lesser affinity, and as such, may not be as efficient in inhibiting CCL2 in humans [106].

### CCL2 inhibition (CNTO888) in metastatic resistant prostate cancer

Administration of CNTO888 has previously demonstrated remarkable anti-tumor effects, both alone and in combination with docetaxel, in murine models of metastatic prostate cancer [66]. Based on these encouraging results, a phase 2 study (NTC00992186) was performed to assess the efficacy of CNTO888 in 46 patients with metastatic resistant prostate cancer; these patients had previously received docetaxel-based chemotherapy and the majority had additional radiotherapy, hormonal therapy or surgery. CNTO888 (15 mg/kg) was administered intravenously every two weeks for the duration of the study. CNTO888 treatment did not result in any complete or partial responses, and only 34% of patients maintained stable disease for more than 3 months. Furthermore, none of the patients showed more than 50% reduction in PSA value. Although CCL2 levels were initially suppressed following treatment, CCL2 concentration quickly increased, surpassing pre-treatment serum concentration, and elevated levels were sustained even after subsequent CNTO888 administration [108].

Taken together, CNTO888 was generally well tolerated with few mild-to-moderate adverse events. Nevertheless, these trials have consistently indicated that CNTO888 is ineffective at sustaining CCL2 blockade in humans. It is important to point out, however, that CCL2 inhibition using the CNTO888 antibody has shown robust anti-tumor responses in several pre-clinical cancer models. Indeed, pre-clinical results were so promising that a phase 2 study was initially planned in ovarian cancer [109] but given the current findings, further development of CNTO888 in oncology needs to be reviewed.

### CCR2 inhibition (MLN1202) in bone metastases

A different approach to CCL2/CCR2 interference was blocking the cognate CCR2 receptor. A humanized IgG1 antibody developed by Millennium Pharmaceuticals, MLN1202, was shown to be specific for the CCR2 receptor, and has been previously tested in several inflammation-related diseases including rheumatoid arthritis, multiple sclerosis, chronic obstructive pulmonary disorder and atherosclerosis with varying results; Phase 2 clinical trials of MLN1202 in multiple sclerosis [110] and in atherosclerosis [111] had moderate success but its use in rheumatoid arthritis showed no clinical improvement [112, 113].

A phase 2 clinical trial (NCT01015560) was conducted to establish the efficacy of MLN1202 in 44 patients with bone metastases resulting from unspecified solid tumors. MLN1202 (8mg/kg) was administered intravenously as a monotherapy on days 1, 15 and 29, and several primary and secondary measures were assessed including uNTX, anti-tumor and immune responses, the latter two which were defined by tumor cell proliferation and myeloid cell infiltration. Similar to CCL2 inhibition by CNTO888, administration of MLN1202 was well tolerated in patients with < 8% reporting serious adverse events although only 14 patients (~32%) had a considerable reduction in uNTX values after 43 days of treatment. Effects of MLN1202 on anti-tumor and immune responses were not disclosed [114].

## CONCLUSIONS

CCL2/CCR2 signaling was demonstrated to play crucial roles in the metastatic process, stimulating cancer cell proliferation, invasion and migration, and promoting metastatic outgrowth and colonization. Experimental evidence from various pre-clinical cancer models have been encouraging and indicate that CCL2 and CCR2 are attractive targets for intervention of metastatic diseases. However, thus far, therapeutics aimed at interference of this chemokine receptor pair have had disappointing results in the clinic.

In depth analysis of the data from clinical trials could shed light into how we can tailor CCL2 or CCR2 blockade to be more effective. Pharmacodynamic studies of the CCL2 inhibitory antibody CNTO888 showed that CCL2 expression was augmented in response to the initial CCL2 inhibition, likely due to a homeostatic feedback mechanism. Indeed, the fact that targeting this pathway seems to have no considerable or long term benefits either in solid tumors or metastatic cancers suggest that other compensatory mechanisms may be involved; whether this is due to increased CCL2, upregulation of CCR2, or contribution by other functionally analogous chemokines and/or chemokine receptors is yet to be delineated. Moreover, it is also important to establish whether inhibition of CCL2 had distinct effects compared to CCR2 inhibition, and if interference of both can lead to better anti-tumor responses in clinical trials.

Overall, this brings into question the translationability of previous pre-clinical data, and further highlights our incomplete understanding of the CCL2-CCR2 signaling network, which is undeniably far more complex than what we initially surmised. Interestingly, a recent study indicated that targeting CCL2-CCR2 signaling may provoke unexpected adverse effects. Cessation or interruption of CCL2 inhibition was shown to increase metastases and accelerate death in four mouse models of metastatic breast cancer. This effect was attributed to augmented cancer cell mobilization from the tumor, increased blood vessel formation and enhanced proliferation of metastatic cells [115]. This is in keeping with the observation in a prostate cancer metastasis model that CCL2 inhibition must be sustained to maintain tumor regression [66], and further questions the effects of CNTO 888 or MLN1202 treatment withdrawal in clinical trials. Taking all these findings into account, it is clear that we may still have a long way to go before we can successfully design chemokine or chemokine receptor-targeted therapies that will profoundly alleviate metastatic cancers.
